# Combination of locoregional radiotherapy with a TIM-3 aptamer improves survival in diffuse midline glioma models

**DOI:** 10.1172/jci.insight.175257

**Published:** 2024-08-15

**Authors:** Iker Ausejo-Mauleon, Naiara Martinez-Velez, Andrea Lacalle, Daniel de la Nava, Javier Cebollero, Helena Villanueva, Noelia Casares, Javier Marco-Sanz, Virginia Laspidea, Oren Becher, Ana Patiño-García, Sara Labiano, Fernando Pastor, Marta M. Alonso

**Affiliations:** 1 Health Research Institute of Navarra (IdiSNA), Pamplona, Navarra, Spain.; 2 Solid Tumor Program, Center for the Applied Medical Research, Pamplona, Navarra, Spain.; 3 Department of Pediatrics, Clínica Universidad de Navarra, Pamplona, Spain.; 4 Center for Cancer Cell Therapy, Stanford Cancer Institute, Stanford University School of Medicine, Stanford, California, USA.; 5 Molecular Therapeutics Program, Center for Applied Medical Research, CIMA, University of Navarra, Pamplona, Spain.; 6 Research Department of Hematology and Oncology, University College London, London, UK.; 7 Jack Martin Fund Division of Pediatric Hematology-Oncology, Mount Sinai, New York, New York, USA.

**Keywords:** Oncology, Therapeutics, Brain cancer, Cancer immunotherapy, Radiation therapy

## Abstract

Pediatric diffuse midline gliomas (DMG) with altered H3-K27M are aggressive brain tumors that arise during childhood. Despite advances in genomic knowledge and the significant number of clinical trials testing new targeted therapies, patient outcomes are still poor. Immune checkpoint blockades with small molecules, such as aptamers, are opening new therapeutic options that represent hope for this orphan disease. Here, we demonstrated that a TIM-3 aptamer (TIM-3 Apt) as monotherapy increased the immune infiltration and elicited a strong specific immune response with a tendency to improve the overall survival of treated DMG-bearing mice. Importantly, combining TIM-3 Apt with radiotherapy increased the overall median survival and led to long-term survivor mice in 2 pediatric DMG orthotopic murine models. Interestingly, TIM-3 Apt administration increased the number of myeloid populations and the proinflammatory CD8-to-Tregs ratios in the tumor microenvironment as compared with nontreated groups after radiotherapy. Importantly, the depletion of T cells led to a major loss of the therapeutic effect achieved by the combination. This work uncovers TIM-3 targeting as an immunotherapy approach to improve the radiotherapy outcome in DMGs and offers a strong foundation for propelling a phase I clinical trial using radiotherapy and TIM-3 blockade combination as a treatment for these tumors.

## Abstract 2

Radiotherapy and TIM-3 blockade for DMG treatment

## Graphical abstract

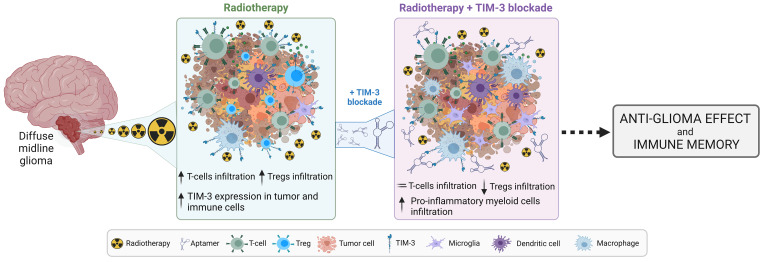

## Introduction

diffuse midline glioma (DMG) is an aggressive brain tumor and the leading cause of pediatric death caused by cancer (1). DMGs are defined as an infiltrative high-grade glioma located in the brain midline (usually the brainstem, spinal cord, cerebellum, or thalamus) with astrocytic differentiation and K27M mutation in either H3.3 (H3F3A) or H3.1 (HIST1H3B/C) (2). However, despite great strides in the understanding of this disease, survival outcomes after treatment are dismal. The standard of care has not changed for more than 50 years, with focal radiotherapy (RT) being the main treatment for DMG (3). RT is not curative, providing tumor stabilization and temporary reduction of symptoms and extending the average survival to approximately 3 months (3, 4). This overall lack of response to traditional treatments including chemotherapeutic agents, target therapies, and RT underscores the need for new therapies targeting the unique biology of DMG tumor microenvironment (5).

Immune checkpoint therapy has changed the treatment paradigm of several advanced cancers (6). Since the first monoclonal antibody against CTLA-4 was approved by the FDA in 2011, it has been followed by many other immune checkpoint inhibitor molecules such as PD-1, PD-L1, LAG-3, and TIM-3 (7). TIM-3 is emerging as another relevant immune checkpoint. TIM-3 was first identified on the surface of T helper 1 cells (Th1) as an immunosuppressive molecule (8). The TIM-3 protein is characterized by having a conserved extracellular IgV domain with N-linked glycosylation sites, followed by a mucin domain that contains O-linked glycosylation. It also has a transmembrane domain and an intracellular cytoplasmic tail with 5 tyrosines (9). TIM-3 expression regulates, by various mechanisms, the function of CD4 Th1 (10), Tregs (11), and CD8 (12, 13) adaptive immune cells. Additionally, TIM-3 is expressed in other immune innate populations such as NK cells (14), DCs (15), macrophages (16), and microglia (17) in the tumor microenvironment. Moreover, TIM-3 was detected on tumor cells in tissues from different patients with cancers such as nasopharyngeal carcinoma (18), melanoma (19), osteosarcoma (20), and others (16, 21–25).

In the last years, aptamers have emerged as a therapeutic alternative to antibody therapy (26). Aptamers are single-strand oligonucleotide ligands that bind to their target with affinity and specificity similar to antibodies. However, they pose some differences: aptamers can be easily produced via in vitro transcription or at a higher cost by direct chemical synthesis, while antibodies are cell-based products (27). Oligonucleotides are immunologically less prone to induce antidrug antibodies (ADA), which is a current problem with many types of monoclonal antibodies hindering the efficacy of the treatment after repetitive administration. Aptamers are smaller, and although they display a shorter half-life, they could likely get deeper into the target tissue than antibodies. All of the above features uncover oligonucleotide-based therapy as a potential therapeutic tool for solid tumors, including brain tumors (28).

In this study, we showed that targeting TIM-3 with a 2F-pyrimidine-RNA oligonucleotideaptamer (Apt1) (29) in combination with locoregional RT (which is the standard of care for DMGs) resulted in a significant antitumor effect accompanied by immune memory acquisition in immunocompetent murine glioma models.

## Results

### TIM-3 local targeting in DMGs with an inhibitory aptamer enhances tumor immunity with limited antitumor effect.

**Figure 1:**
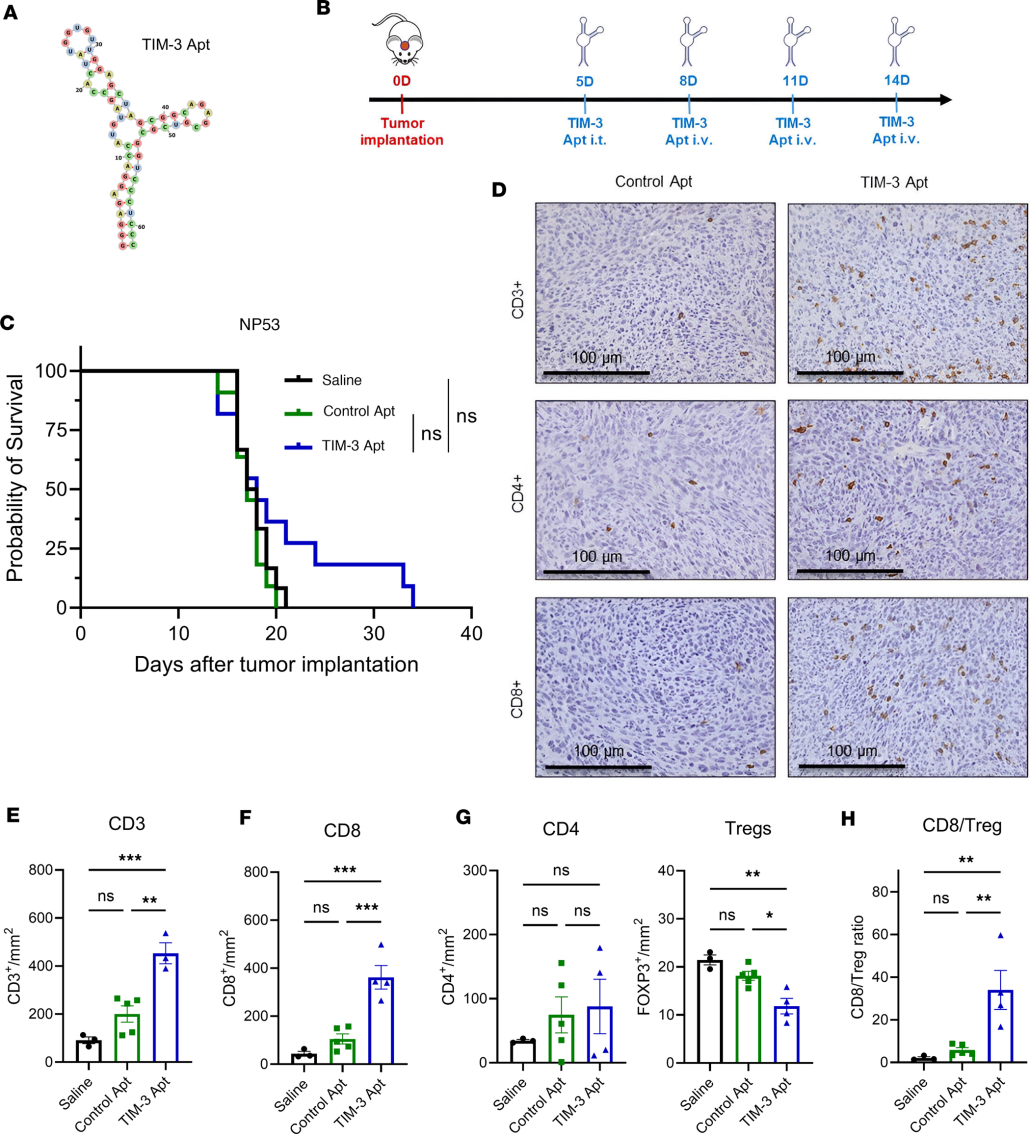
Evaluation of the antitumor effect of anti-TIM-3 oligomeric aptamer blockade. (**A**) 2-dimensional predicted structure of the Apt1 aptamer antagonist of TIM-3. (**B**) Schedule of survival experiments performed with murine NP53 DMG cells. Cells were implanted on day 0. On Day 5, 380 pmol/mouse of Apt1 or Control Apt was administered intratumorally (i.t.), and on days 8, 11, and 14, each of the aptamers (32 0pmol/mouse) was administered intravenous (i.v.). (**C**) Kaplan–Meier survival curves of mice bearing NP53 (*n* = 12 per group) DMG cells treated with TIM-3 Apt or control Apt. (**D**) CD3, CD4, and CD8 immune-staining representative images after the indicated treatments. Scale bars: 100μm. (**E**–**G**) CD3^+^ (**E**), CD8^+^ (**F**), CD4^+^, and FOXP3^+^ Tregs (**G**) cell infiltration per mm^2^ of NP53 tumors. Graph showing the quantification of positive cells infiltrating the brain at sacrificed day after the indicated treatments per mm^2^ (Saline, control Apt, and TIM-3 Apt; *n* = 3–5). **(H)** Analyses of the CD8^+^ cell/Treg proinflammatory ratio in the tumor microenvironment (*n* = 3–5). Data were analyzed with 1-way ANOVA. Bar graphs indicate the mean ± SEM (*P < 0.05; **P < 0.01; ***P < 0.001; ****P < 0.0001).

First, we evaluated the therapeutic value of TIM-3 antagonist monovalent aptamer (Apt1) as monotherapy in a DMG preclinical immunocompetent model. Apt1 is a short 62 nucleotide RNA 2F-Py modified oligonucleotide (Figure 1A) that binds to TIM-3 and counteracts the TIM-3 inhibitor signal (29). We have previously demonstrated that intratumoral administration of an anti-TIM-3 monoclonal antibody into the brainstem is more efficacious than a single systemic administration due to the integrity of the brain-blood barrier (BBB) in this disease (30). In fact, in the context of DMGs, alternative routes of administration have been evaluated in the clinic, such as Convention Enhanced Delivery (CED) or direct injection of therapeutic agents such as oncolytic viruses for these tumors (31); however, giving more than a single administration in the clinic harbored its challenges due to the risk of accessing the brainstem (31, 32). Thus, mice bearing orthotopical NP53 DMG cells were treated with a single intratumoral injection of Apt1 (380 pmol/mouse; 5 days after implantation) or its corresponding controls (Saline and control Apt group) and with 3 subsequent intravenous doses (8, 11, and 14 days after tumor implantation; 320 pmol/mouse) (Figure 1B). Under this experimental setting, Apt1 treatment as monotherapy did not result in a statistically significant increase in the median survival of NP53 tumor–bearing mice (Figure 1C). To elucidate if the TIM-3 Apt1 treatment affected the immune infiltrate in the tumor microenvironment, we characterized the adaptive immune populations at the endpoint by IHC (Figure 1D). Apt1-treated mice showed a significant increase in the number of CD3^+^ T cells compared with control Apt and saline-treated groups (Figure 1E). This increase in T cells in the Apt1-treated mice was due to a significant increase in the number of CD8^+^ lymphocytes (Figure 1F). No differences in the number of total CD4^+^ T lymphocytes in the tumor of treated mice were observed. However, there was a drastic reduction in the number of Tregs (Foxp3^+^) in the Apt1-treated group compared with the control mice (Control Apt and saline groups; Figure 1G). Apt1 treatment significantly increased the proinflammatory CD8^+^/Foxp3^+^ T cell ratio (Figure 1H). These results indicate that Apt1 treatment causes an increase in the number of CD8^+^ T cells and a reduction of Tregs in the TME, but this is insufficient to promote a robust antitumor effect in the DMG model.

### TIM-3 blockade with Apt1 promotes the release of effector cytokines.

**Figure 2:**
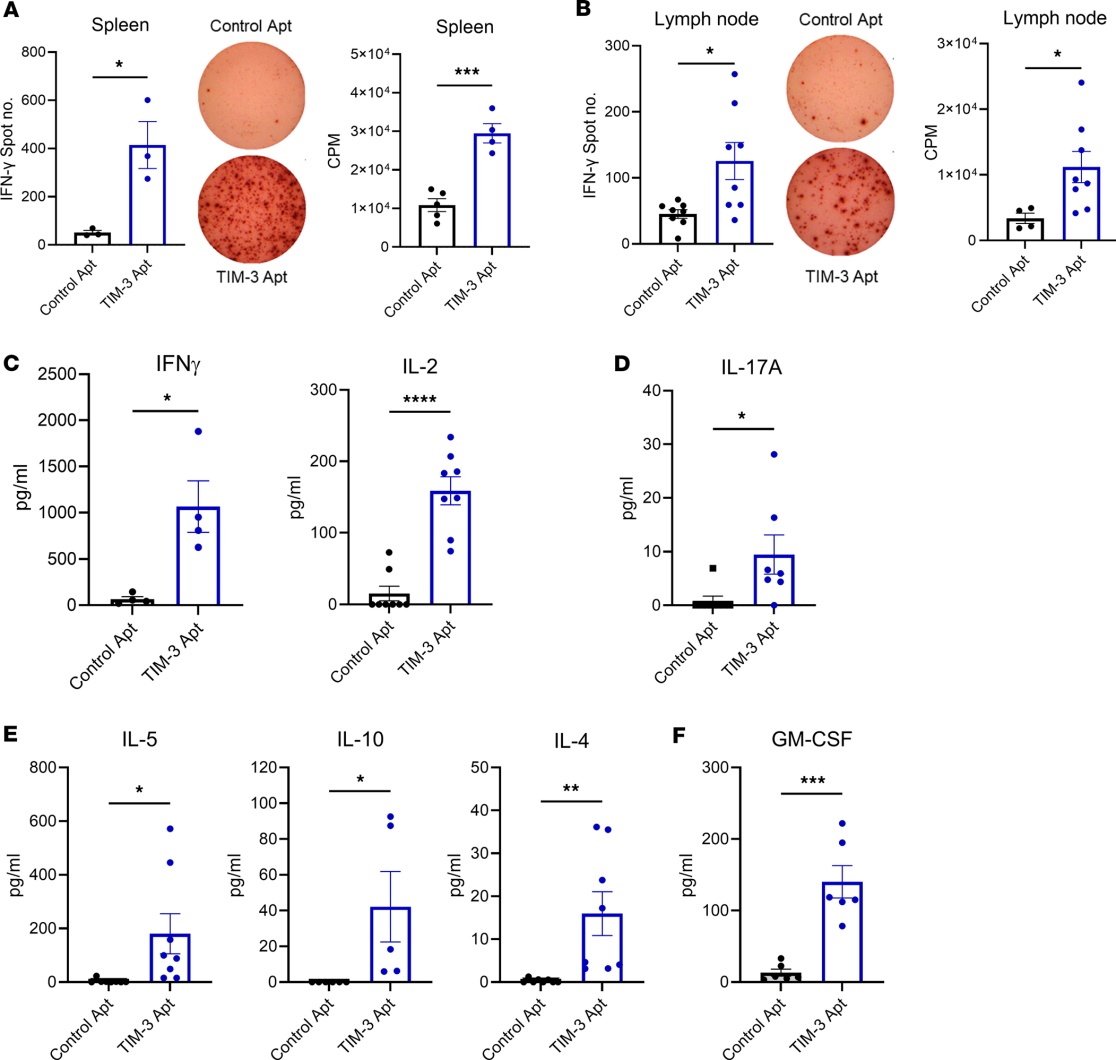
Characterization of effector cytokines produced by TIM-3 blockade. (**A** and **B**) ELISPOT analyses of IFN-γ spot number and CPM of (**A**) splenocytes and (**B**) lymph nodes 15 days after tumor implantation (*n* = 3–8). Image shows one well of a 96-well plate. (**C**) MACSplex analysis of IL-2 (*P* < 0.0001) and IFN-γ (*P* = 0.0119) proinflammatory Th1 cytokines 15 days after tumor implantation between Control Apt and TIM-3 Apt groups (*n* = 4–8). (**D**) MACSplex analysis of IL-17A (*P* = 0.029) Th17 cytokines 15 days after tumor implantation between saline and TIM-3 Apt groups (*n* = 7). (**E**) MACSplex analysis of IL-4 (*P* = 0.0082), IL-5 (*P* = 0.032), and IL-10 (*P* = 0.042) Th2 characteristics cytokines 15 days after tumor implantation between saline and TIM-3 Apt groups (*n* = 5–8). (**F**) MACSplex analysis of GM-CSF (*P* = 0.0003) innate immune response related cytokine 15 days after tumor implantation between saline and TIM-3 Apt groups (*n* = 6). Data were analyzed with a student *t* test. Bar graphs indicate the mean ± SEM (*P < 0.05; **P < 0.01; ***P < 0.001; ****P < 0.0001).

To determine whether Apt1 could enhance the endogenous specific effector immune response against NP53 antigens, we performed IFN-γ ELISPOT and ^3^H thymidine proliferation assays to study the importance of the T cell systemic immune response in a distant secondary lymphoid organ (spleen) or proximal ones (lymph nodes) to the tumor. We detected a significant increase in IFN-γ spot number measured by ELISPOT as well as higher T proliferative rates measured by ^3^H thymidine (CPM) in the splenocytes of animals treated with Apt1 compared with the control Apt group (Figure 2A). We performed similar experiments with lymphocytes from tumor-draining lymph nodes, and we also observed significant differences between the different treatment groups (Figure 2B). To better assess the type of immune response elicited by TIM-3 Atp1 treatment, we performed a cytokine MACSplex analysis. Splenocytes from Apt1-treated mice secrete significantly higher concentrations of cytokines IL-2 and IFN-γ, indicative of an effector-activated Th1 response (Figure 2C). In addition, T cells also produced higher levels of IL-17A associated with a Th17 response (Figure 2D). However, Apt1 treatment also led to a higher Th2 response, as cytokines IL-4, IL-5, and IL-10 were induced upon Apt1 treatment (Figure 2E). Interestingly, Apt1 increased the production of GM-CSF (Figure 2F), which functions in the recruitment and activation of myeloid cells, including dendritic cells and microglia. Taken together, these data suggest that TIM-3 targeting promotes a multipronged immune response activating helper effector arms of the immune system. This is probably due to the ubiquitous expression of TIM-3 in all the different immune cell types.

### RT increases TIM-3 expression in DMGs.

**Figure 3:**
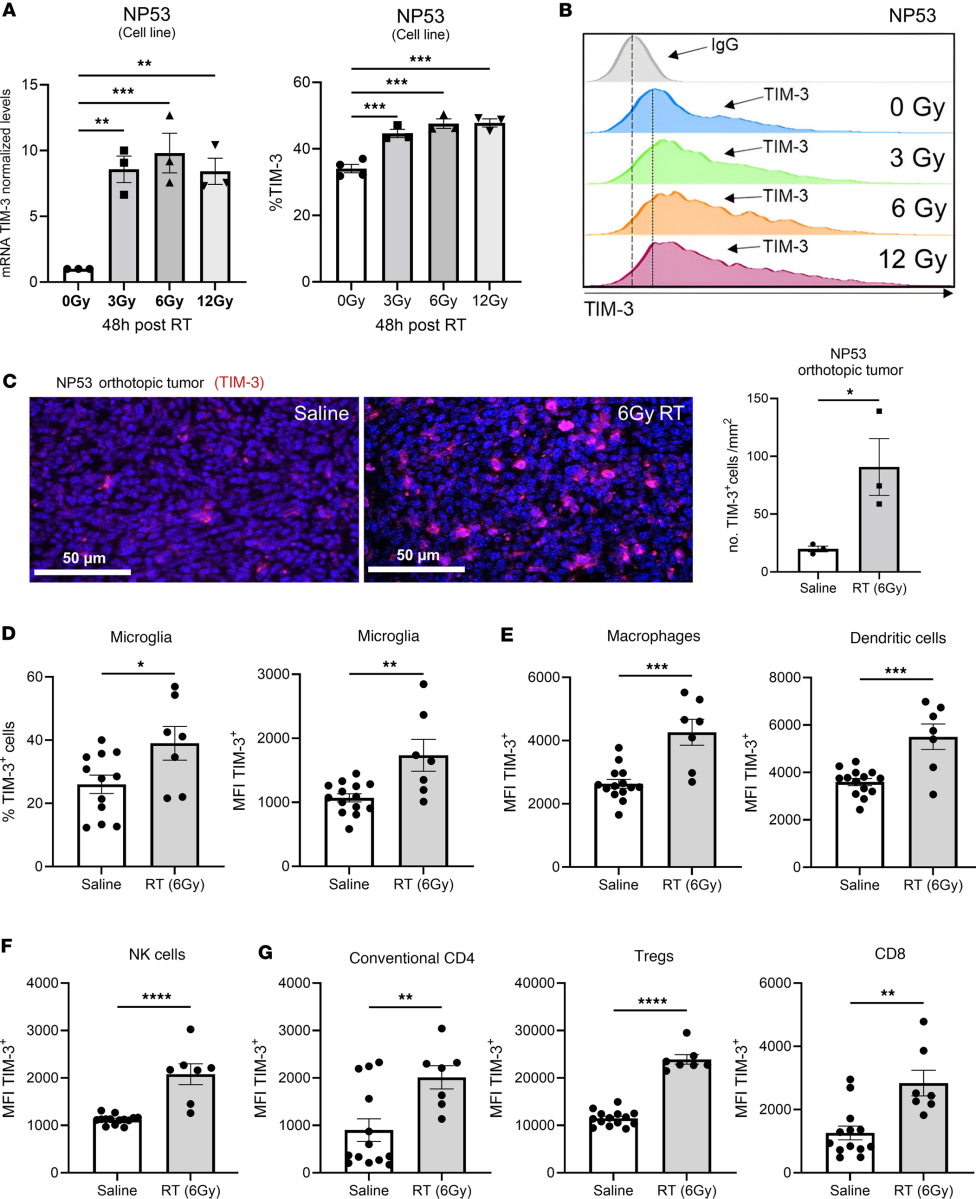
Assessment of TIM-3 expression after RT treatment. (**A**) Real-time PCR analysis of TIM-3 expression in NP53 tumors cells 48 hours after RT (*n* = 3). (**B**) Flow cytometry analysis of TIM-3 expression in NP53 tumor cells 48 hours after 3, 6, and 12 Gy of RT (*n* = 3). (**C**) *Left*, a representative image of TIM-3 expression analysis in mice bearing NP53 tumor cells comparing saline and RT by IF. *Right,* analysis of TIM-3^+^ cells per mm^2^ (*n* = 3). Scale bars: 50μm. (**D**) *Left,* flow cytometry analysis of the percentage of microglia cells that expressed TIM-3. *Right,* MFI of TIM-3 on microglia cells after RT (6 Gy) in a DMG orthotopic model (*n* = 7–12). (**E**–**G**) Flow cytometry analysis of TIM-3 expression MFI on (**E**) macrophages, dendritic cells, (**F**) NK cells, (**G**) conventional CD4, Tregs, and CD8 T cells after RT (6 Gy) in a DMG orthotopic model (*n* = 7–12). Data were analyzed with 1-way ANOVA (**A**, **B**, **D**–**G**) and student *t* test (**C**). Bar graphs indicate the mean ± SEM (*P < 0.05; **P < 0.01; ***P < 0.001; ****P < 0.0001).

Because RT is the standard of care for DMG, next, we evaluated the effect of this approach on TIM-3 expression. It has been previously described that TIM-3 was expressed in different tumor types, including DIPGs (30) and gliomas, although at low levels (33), and its expression could be associated with hypoxia in brain damage responses (34). Thus, we wanted to determine whether RT would increase TIM-3 expression, creating a potential synergistic effect when combined with TIM-3–targeting agents. We irradiated NP53 DMG cells with different Gys (3, 6, and 12), and we evaluated TIM-3 expression at the RNA and protein levels at different time points, 24 and 48 hours, after RT. At the RNA level, we did not observe an increase in the expression of TIM-3 at 24 hours after RT (Supplemental Figure 1A; supplemental material available online with this article; https://doi.org/10.1172/jci.insight.175257DS1). Importantly, at 48 hours after RT, the cells displayed a significant increase in TIM-3 expression in both mRNA (Figure 3A) and protein (Figure 3B) levels at all the doses. Of importance, TIM-3 expression was significantly increased in a DMG orthotopic model after 6 Gy RT treatment compared with the control measured at mRNA (Supplemental Figure 1B) and protein levels (Figure 3C). Interestingly, after RT, TIM3 expression increased (percentage and mean fluorescence intensity) in several immune populations of the TME including microglia (Figure 3D), macrophages, dendritic cells (Figure 3E), NK cells (Figure 3F), conventional CD4, Tregs, and CD8 (Figure 3G). No differences were found in the expression of TIM-3 in monocytes or B cells after RT (Supplemental Figure 1D). These data demonstrate that TIM-3 expression in DMG is induced after RT and opens the possibility of enhancing its therapeutic window with the combination of an anti-TIM-3 agent.

### Apt1 TIM-3 blockade enhances the antitumor RT efficacy in DMG models.

**Figure 4:**
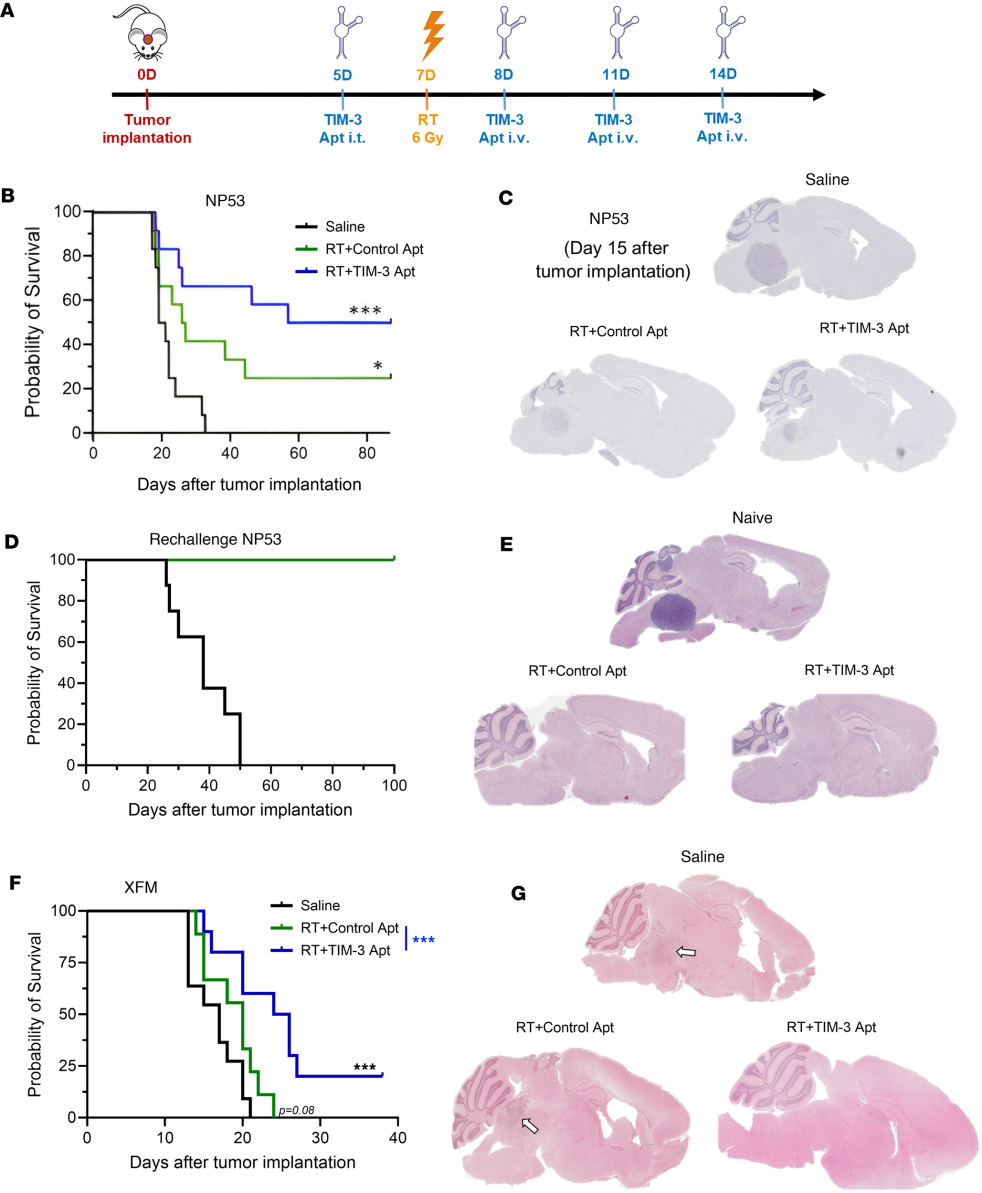
Evaluation of the antitumor effect of RT and Apt1 TIM-3 blockade combination in DMG models. (**A**) Schedule of survival experiments performed with murine NP53 and XFM DMG cells. Cells were implanted on day 0. On Day 5, 380 pmol/mouse of Apt1 or control Apt was administered intratumorally (i.t.), and on days 8, 11, and 14, each of the aptamers (320 pmol/mouse) was administered intravenous (i.v.). 6 Gy of locoregional RT was performed 7 days after tumor implantation (2 days after the first dose of TIM-3 Apt). (**B**) Kaplan-Meier survival curves of mice bearing NP53 (*n* = 12 per group) cells treated with saline, RT + Control Apt, and RT + TIM-3 Apt. (**C**) Representative image of NP53 tumors harvested from mice 15 days after tumor implantation on indicated groups. (**D**) The long-term survivors in both RT groups from (**B**) were rechallenged with NP53 cells and compared with control naive mice (*n* = 8). (**E**) Representative H&E staining images of NP53 tumors harvested from mice at the time of death (naive) or at the endpoint of the experiment (long-term survivors) on indicated groups. (**F**) Kaplan-Meier survival curves of mice bearing XFM (*n* = 10 per group) cells treated with saline, RT + Control Apt, and RT + TIM-3 Apt. (**G**) Representative image of XFM tumors harvested from mice on indicated groups (*P < 0.05; **P < 0.01; ***P < 0.001; ****P < 0.0001).

To determine whether locoregional RT could synergize with TIM-3 aptamer, we perform locoregional RT in combination with TIM-3 Apt1 in 2 different immunocompetent orthotopic aggressive DMG models (NP53 and XFM). Mice were treated with an intratumoral neoadjuvant dose of Apt1 (380 pmol/mouse) 2 days before RT and then with 3 intravenous subsequent doses (320 pmol/mouse; Figure 4A). Treatment with RT resulted in a significant increase in the median survival in both RT groups (RT + control apt and RT+ TIM-3 Apt-1) compared with saline and led to long-term survival in mice bearing NP53 tumors. We observed a significant increase in median survival (30 days RT + control Apt versus 58 days RT+TIM-3 Apt) with the appearance of a considerable number of long-term survivors (25% RT + control apt versus 50% RT + TIM-3 Apt) (Figure 4B) indicating a potential synergistic effect between RT and TIM-3 targeting by Apt1. Mice sacrificed 15 days after tumor implantation showed that the tumor size of groups treated with RT and the combination of RT and TIM-3 Apt was significantly smaller (Figure 4C). Then, we performed a rechallenge experiment to ascertain whether treated mice developed antiglioma immune memory. We observed that 100% of the mice cured from the treatment were protected from a rechallenge with a lethal dose of glioma cells, indicating the existence of an immune memory in the treated mice (Figure 4D). In agreement with these data, anatomopathological analyses of the brains showed that long-term survivors were free of tumors (Figure 4E). Treatment of mice bearing XFM tumors with the combination treatment also led to an increase in median survival compared with the saline group (Saline = 17 days versus RT + TIM-3 Apt = 25 days; *P* = 0.002) and RT + control Apt (20 days; *P* = 0.016), leading to 20% long-term survivors (Figure 4F). In addition, mice sacrificed 10 days after tumor implantation demonstrated that the tumor size of groups treated with RT and the combination of RT and TIM-3 Apt was significantly smaller (Figure 4G). Overall, our data support the value of targeting TIM-3 with an aptamer in combination with RT in DMG.

### A combination of RT and anti-TIM-3 aptamer results in an increase in immune infiltration in the tumor microenvironment.

**Figure 5:**
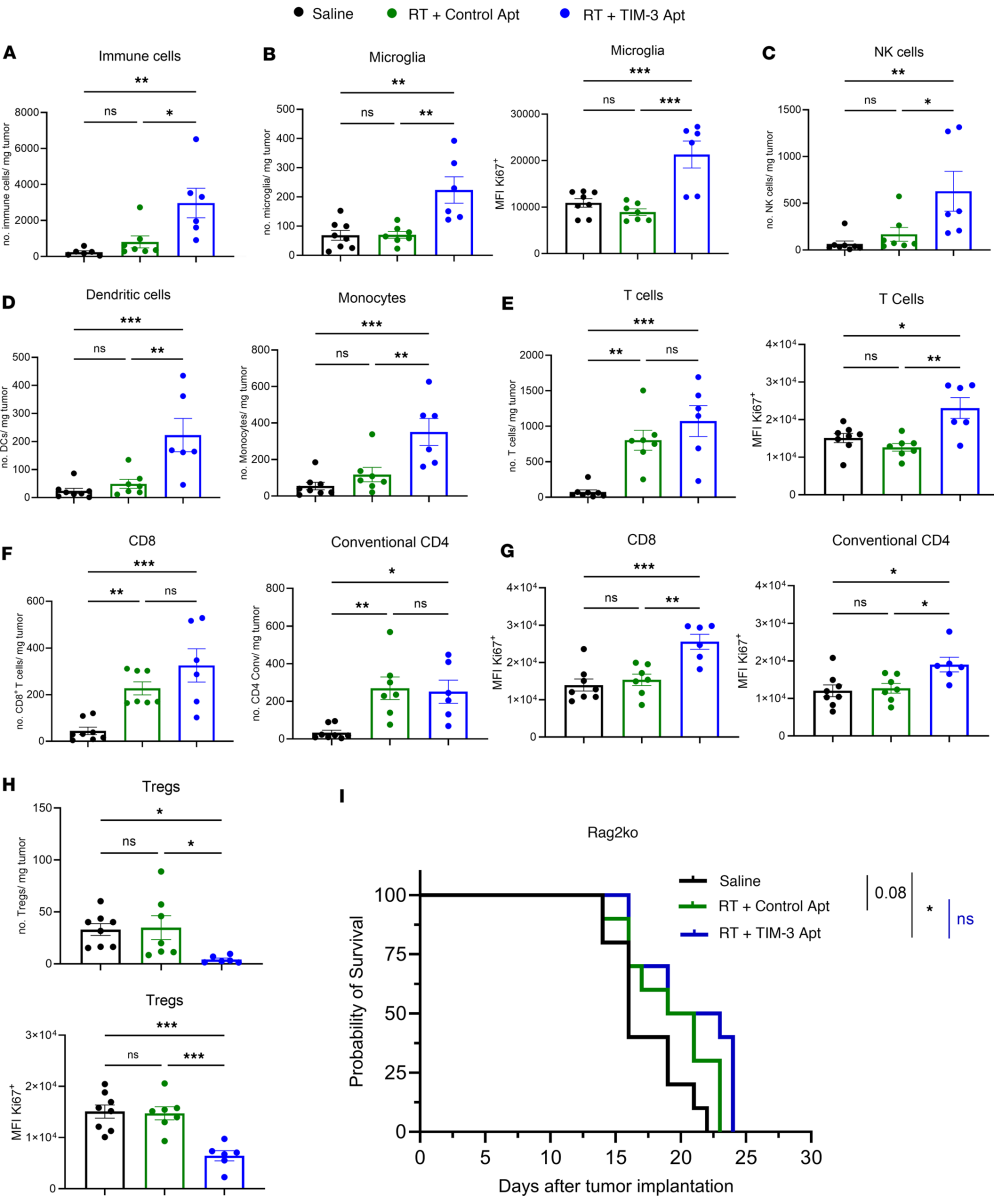
Characterization of innate and adaptive immune response modulation by RT and TIM-3 blockade combination. NP53 cells were engrafted, and animals were treated with saline (*n* = 8), RT (6 Gy) control Apt (*n* = 7), or RT and TIM-3 (*n* = 6). Animals were sacrificed 15 days after tumor implantation and 8 days after RT. (**A**) Flow cytometry analyses of immune cells (CD45^hi^) per mg of tumor on the indicated groups. (**B**) *Left*, flow cytometry analyses of microglia cells (CD45^med^CD11b^+^) per mg of tumor on the indicated groups. *Right*, quantification of Ki67^+^ MFI on microglia cells by flow cytometry. **(C)** *Left*, flow cytometry analyses of dendritic cells per mg of tumor. *Right*, flow cytometry analyses of monocytes per mg of tumor on the indicated groups. (**D**–**F**) Flow cytometry analyses of (**D**) NK cells, (**E**) T cells, and (**F**) CD8, conventional CD4, and Tregs per mg of tumor. (**G** and **H**) Quantification of Ki67^+^ MFI on (**G**) T cells including CD8, conventional CD4, and (**H**) Tregs cells by flow cytometry. (**I**) Kaplan-Meier survival curves of Rag3-KO mice bearing NP53 (*n* = 10 per group) cells treated with saline, RT + Control Apt, and RT + TIM-3 Apt. Data were analyzed with 1-way ANOVA. Bar graphs indicate the mean ± SEM (*P < 0.05; **P < 0.01; ***P < 0.001; ****P < 0.0001).

We analyzed tumor immune infiltration to better understand the immune mechanism that underlines the antitumor efficacy of the RT and TIM-3 Apt combo. NP53 tumor cells were implanted orthotopically, and the mice were randomized to either of the 3 groups of treatment (Saline, RT + control Apt, and RT + TIM-3 Apt1). Mice were sacrificed 15 days after the implantation of the tumor following the same schedule as in the survival experiment (Figure 4A), and immune populations were analyzed by flow cytometry (Figure 5) and IHC (Supplemental Figure 2). Further, RT and TIM-3 combo led to an increase in the number of total immune cells (CD45^hi^) per mg of tumor (Figure 5A). In addition, we observed a significant increase in the number and proliferative state measured by Ki67 of microglia (CD45^med^CD11b^+^) compared with the other groups (Figure 5B). RT and TIM-3 Apt combination also increased the number of NK and B cell (Figure 5C and Supplemental Figure 2B) innate immune populations compared with the other 2 treatment groups.

Regarding the myeloid cells, the combination increased the number of monocytes and dendritic cells (Figure 5D); however, no differences were found in the number of macrophages (Supplemental Figure 2A) in the tumor microenvironment. Locoregional RT–treated mice (both groups) displayed a significant increase in the accumulation of proliferative T cells per mg of tumor compared with saline-treated brains (Figure 5E). We observed a significant increase in the number of conventional CD4^+^ and CD8^+^ T cells in both RT groups (Figure 5F). However, although the number of T cells, including conventional CD4^+^ and CD8^+^ lymphocytes, remained constant between the RT groups, we only saw a significant increase in the proliferative status in the combination group (Figure 5G). Moreover, we observed a substantial decrease in the Treg population and its proliferative capacity in the RT-TIM-3 Apt1 treated group (Figure 5H). Only combination treatment significantly increased the proinflammatory CD8^+^ T cell/Treg and CD4^+^ T cell/Treg ratios at day 15 after tumor implantation (Supplemental Figure 2C). We confirmed these results by IHC (Supplemental Figure 2, D and E). Additionally, the serum cytokine analysis performed 15 days after implanting the cells in the NP53 model did not reveal any significant difference (Supplemental Figures 3, and B). To further rule out the roles of the T cells in therapeutic efficacy, we used immunodeficient Rag2^–/–^ mice, which lack functional T cells but have macrophage and microglial populations. Interestingly, survival studies demonstrated a significant loss of the therapeutic effect in the combination groups (Figure 5I). We still observed a significant impact in mice treated with RT + TIM-3 Apt, probably due to the effect of RT on tumor cells and TIM-3 blockade on myeloid cells (Figure 5I). All these data suggest that myeloid cells, T cells, and Tregs could be one of the main mechanisms of action of Apt1 by modifying the tumor microenvironment and counteracting the immunosuppression mediated by both populations (33).

## Discussion

DMG’s meager survival rate has not changed despite the combination of RT with targeted therapies (5), emphasizing the urgent need for effective treatments. Immune-checkpoint blockade therapy, including anti-CTLA-4 and PD(L)-1 antibodies, is the most successful immunotherapy approach in many patients with cancer (35). Nonetheless, patients with brain tumors remain elusive to this type of treatment (36, 37), probably due to the unique, highly immunosuppressive immune tumor microenvironment of these types of tumors (38). In this work, we show that targeting the TIM-3 axis is a vulnerability to DMG tumors, favoring the induction of a potent systemic antitumor immune response that can be efficacious in controlling tumor progression when combined with locoregional RT. TIM-3 is an attractive target for cancer immunotherapy (39) due to its expression in cells of the adaptive (40, 41) and innate (9, 42) immune systems, including the tumor cells (43–45).

TIM-3 blockade by oligomeric aptamers has already been shown to control tumor growth in murine models of CT26, alone or combination with a PD-1 antibody, by significantly increasing the proinflammatory CD8/Treg ratio in the tumor microenvironment (46). In the current orthotopic glioma models, TIM-3 targeting with Apt1 also considerably increased the CD8/Treg ratio. The therapeutic effect of inhibiting TIM-3 in monotherapy seems to be modest. Still, TIM-3 Apt1 treatment promotes a pool of effector cytokine secretion activating all the effector arms of the immune system, including a Th2 immune response. The increase of IL-10 after treatment intrigues us because it is overexpressed in patients with glioblastoma multiforme (GBM) (47) and it is associated with increased glioma cell proliferation and invasion in preclinical models (48). Additionally, IL-10 has been shown as one of the main cytokines with antiinflammatory effects in human tumors due to its ability to suppress T cells (49). This upregulation of IL-10 may dilute the proinflammatory effect of Apt1, decreasing the therapeutic effect of TIM-3 blockade and opening the possibility of combining IL10 blockade with anti-TIM-3 agents to improve the therapeutic outcome in future studies. RT is the standard of care for DMG (3) and also a fundamental requirement in any first-line DMG clinical trial (31, 32), so we decided to combine our TIM-3 Apt with locoregional RT. TIM-3 blockade using a monoclonal antibody in combination with RT and anti-PD-1 has already shown promising efficacy in preclinical models of GBM (50), although other molecules such as aptamers have never been used. Moreover, the combination in DMG models of RT and TIM-3 blockade had never been tested. In our work, the antitumor effect of a RT and TIM-3 aptamer combination was remarkable, increasing the median overall survival of treated mice associated with higher infiltration of proinflammatory immune cell populations in the TME. RT has been described as capable of increasing the infiltration of T cells into the microenvironment of brain tumors (51). However, RT not only causes the infiltration of proinflammatory T cells but also increases the infiltration of Tregs (52). In brain tumors, the percentage of tumor-infiltrating Tregs in patients is strongly correlated with the WHO grade. It demonstrates that the accumulation of Tregs in glioblastomas contributes to the dismal immune responses observed in these tumors (53). Interestingly, treatment with TIM-3 Apt1 after RT causes a significant decrease in the infiltration of Tregs, which is promoted by RT. Therefore, we speculate that TIM-3 targeting may directly affect Tregs due to the importance of this receptor in their phenotype and function (33). Further, our combination treatment also improves the expansion of myeloid cells, according to previous works demonstrating this population’s fundamental role after the TIM-3 blockade (30).

In summary, we provide evidence that the combination of RT and TIM-3 targeting is capable of inducing a significant increase in overall median survival in DMG models. This leads to the expansion of myeloid populations and T cells in the TME and the generation of immune memory. Additionally, we demonstrate the importance of regulatory T cells in TIM-3 aptamer targeting.

## Methods

### Sex as a biological variable.

Our study examined male and female animals in the same proportion; similar findings are reported for both sexes.

### Cell lines and culture conditions.

Murine DMG cell lines XFM and NP53 were provided by Oren Becher (OB) (Jack Martin Fund Division of Pediatric Hematology-Oncology, Mount Sinai, New York, USA). Cell lines were generated from DMG tumors arising in genetically modified mice (54). NP53 cell line was obtained from tumors generated using RCAS (Replication-competent ASLV long terminal repeat with a splice acceptor) system in NP53^fl/fl^ mice, obtained from crossing Nestint tv-a (Ntv-a) and p53 floxed (C57BL/6 background with p53^fl/fl^). Nestin Tv-a mice contain the TVA receptor (receptor for RCAS to infect) under the Nestin promoter. The cells that express Nestin are susceptible to virus infection, producing PDGF-B signaling, p53 loss, and ectopic H3.3-K27 mutation in those cells. The cells that mainly express Nestin are the glial progenitor cells. OB derived the NP53 cell line from tumors generated using this system, and, therefore, we implanted these cell lines in the same mice in which it was created. The XFM cell line was generated in the same way that NP53 from tumors developed in a mouse model driven by PDGF-B signaling and Ink4a and ARF loss with H3 WT. Ink4a-ARF deletions are more common in secondary DMGs induced by RT. Both cell lines were maintained in Dulbecco′s Modified Eagle Medium supplemented with 10% FBS and 1% antibiotics streptomycin/ penicillin. All cells were maintained in a humidified atmosphere containing 5% CO_2_ at 37°C. All cell lines were routinely tested for mycoplasma (Mycoalert mycoplasma detection kit; Lonza) and authenticated at the CIMA Genomic Core Facility (Pamplona, Spain) using DNA profiling.

### Animal studies.

The orthotopic DMG model was engrafted by injection into the pons using a guidescrew system (Taconic Farms Inc.) (55). The NP53 cells (1 × 10^4^ cells) were implanted in transgenic mice provided by OB, and the XFM (1 × 10^3^ cells) were implanted in balb/c mice. The cells were administered in 3 μL of PBS. The animals were randomly assigned to the following 3 groups: control mice injected with saline or aptamer control Apt and mice injected with TIM-3 aptamer (Apt1). Apt1 was administered intratumorally in 3 μL (380 pmol/mouse) and 3 times intravenous (320 pmol/mouse) 5, 8, 11, and 14 days after the cell implantation. In the RT experiments, animals were randomly assigned to the following 3 groups: saline, RT + control apt, and + TIM-3 Apt. 6 Gy of RT was given on day 7, and the aptamer was injected using the same schedule as the previous experiment. In the case of immunocompetent murine models, in which kinetics are very fast, we consider long-term survivors to be animals that live at least 3 times longer than the median survival of the control animals. For rechallenge experiments, mice that survived 3 times longer than the median survival time of the control group were orthotopically reimplanted with the same number of tumor cells in the brain.

### TIM-3 aptamer production.

The Atp1 anti-TIM-3 used in the study was previously described (29) and it is 2F′-pyrimidine RNA aptamer: 5′-GGGAGAGGACCAUGUAGCCACUAUGGUGUUGGAGCUAGCGGCAGAGCGUCGCGGUCCCUCCC-3′. As the control Apt in the experiments, it was used as a randomized 2F′-RNA 25N aptamer flanked containing the constant regions at 5′ and 3′ than the Apt1 TIM-3 aptamer. Both aptamers were produced by transcription from a double-stranded DNA oligonucleotide template generated from hybridization of 2 partially complementary sequences and amplified by PCR with the primers forward: GGGGAATTCTAATACGACTCACTATAGGGAGAGGACCATGTA and reverse: GGGAGGGACCGCGACGCTCTG. The forward primer includes the T7 promoter to allow its transcription using the T7 Durascribe Kit (Epicentre). The aptamers were purified by polyacrylamide gel electrophoresis (PAGE) and refolded by hitting.

### IFN-γ ELISPOT.

NP53 cells were incubated with murine recombinant IFN-γ (100 IU/mL). 24 hours later, splenocytes and lymph nodes were isolated from mice and cocultured with NP53 cells (ratio of 10:1) for 24 hours in a 96-well plate. A mouse IFN-γ ELISPOT set (551083 BD) was used according to the manufacturer’s instructions, and the results were measured using an Immunospot S6 Analyzer (Macro, Immunospot). The results of the IFN-γ ELISPOT were normalized per 1 × 10^4^ cells.

### Flow cytometry.

NP53 single-cell suspensions were stained for flow cytometry. Dead cells were excluded by PromoFluor-840 staining (1:10,000, PK-PF840-3-01). Tumor cells were stained using TIM-3-PE (Biolegend Cat: 119703, Clone: RMT3-23; 1:200). For immune characterization, excised tumors in the experiment were weighted and mechanically dissociated using a scalpel, incubated with collagenase IV/DNase I (17018-029 Gibco/11284932001 Roche) with rotation for 15 minutes, and then incubated twice for 10 minutes at 37°C. The solution was filtered through a 70 mm cell strainer (Thermo Fisher Scientific). After the addition of a 30% Percoll solution (17-0891-01 GE Healthcare), tumor cells were isolated by centrifugation at 500*g* for 20 minutes. Single-cell suspensions were then stained for flow cytometry. Dead cells were excluded by PromoFluor-840 staining (1:10,000, PK-PF840-3-01). Our previous published work lists the fluorochrome-tagged monoclonal antibodies (mAbs) used in this assay (55). Cells were fixed and permeabilized for nuclear staining using BD Cytofix/Cytoperm Plus (555028 BD Biosciences) and then stained according to the manufacturer’s instructions. The remaining samples were then analyzed using CytoFLEX (Beckman Coulter) and FlowJo software (BD Biosciences). The flow gating strategy used for tumor microenvironment immune population characterization experiments was explained in Ausejo-Mauleon et al. (55). Immune population data was normalized against the weight of the tumors after removal from the brain.

### IHC.

The brains were embedded in paraffin blocks, and 3 μm tissue slides were stained using the following antibodies: CD3 (1:300; Clone: SP7, NeoMarkers), CD4 (1:1,000; EPR19514, ab183685 Abcam) CD8a (1:1,000, (D4W2Z) #98941 Cell Signaling) and FoxP3 (1:400; clone JFK-16s, 14–5773 eBiosciences, Thermo Fisher Scientific). The slides were visualized using 3,3-diaminobenzidine (DAB, K346889-26 Dako) and counterstained with hematoxylin (HX85602653 MERCK). The preparations were observed under a confocal microscope (0114107 Nikon Y-THS) and scanned using an Aperio C52 image capture device (Leica Microsystems) and Aperio ImageScope 12.1.0 software (Leica Microsystems).

### MACSPlex cytokine assay.

Splenocytes were isolated from mice and cocultured with NP53 cells (ratio of 10:1) for 24 hours in a 96-well plate. Supernatants were analyzed by the MACSPlex 12 cytokine Kit (Miltenyi Biotec). Serum was collected from mice bearing NP53 tumors 15 days after tumor implantation. All working steps were carried out according to the manufacturer’s instructions, and washing procedures were performed with a centrifuge. Flow analysis was performed using MACS Quant Analyzer (Miltenyi Biotec). Data analysis was performed using Flow Logic (V7.2.1) and BeadLogic (V7) (Miltenyi Biotec).

### RNA extraction and real-time PCR.

Total RNA was extracted from isolated cells using TRIzol according to the manufacturer’s instructions (Life Technologies). RNA samples were quantified using a Nanodrop 1000 spectrophotometer (Thermo Fisher Scientific) and stored at −80°C. A microgram of RNA was reverse transcribed using a high-capacity cDNA reverse transcription kit (Applied Biosystems, Thermo Fisher Scientific). Afterward, cDNA was amplified using SYBR-Green Master Mix (Applied Biosystems). The gene-specific assay was murine TIM-3. HPRT1 was used as the housekeeping control gene, and all samples were run in triplicate. The sequences of the primers for TIM-3 are forward, 5′-TCAGGTCTTACCCTCAACTGTG-3′ and reverse, 5′- GGGCAGATAGGCATTTTTACCA-3′. Real-time PCR was monitored using an ABI 7700 sequence detection system (Applied Biosystems). The fold changes in the expression of the genes of interest were calculated as the mean values calculated using the 2^–ΔΔCT^ method.

### Statistics.

For the in vitro experiments, data are expressed as the mean ± SD, and comparisons were evaluated by the 2-tailed Student’s *t* test or 1-way ANOVA. The effect of TIM-3 Apt and RT, alone or in combination, in glioma orthotopic models was assessed by plotting survival curves using the Kaplan-Meier method. Survival of different treatment groups was compared using the log-rank test. GraphPad software (Prism version 9.3.1) was used for the statistical analysis.

### Study approval.

Ethical approval for the animal studies was granted by the Animal Ethical Committee of the University of Navarra (CEEA; Comité Etico de Experimentación Animal) under the protocol numbers CEEA/069–13. All animal studies were performed at the veterinary facilities of the Center for Applied Medical Research in accordance with institutional, regional, and national laws and ethical guidelines for experimental animal care.

### Data availability.

Data are available in the “Supporting Data Values” file.

## Author contributions

IAM, NMV, MMA, and FP conceptualized and designed the project. IAM, NMV, AL, DDLN, JC, HV, NC, JMS, VL, OB, APG, SL, FP, and MMA were responsible for development of methodology, acquisition of data (provided animals, acquired and managed patients, provided facilities, etc.), writing, reviewing, and/or revising the manuscript, and for administrative, technical, or material support (i.e., reporting or organizing data and constructing databases). FP and MMA supervised the study.

## Supplementary Material

Supplemental data

Supporting data values

## Version 1 08/15/2024

In-Press Preview

## Version 2 09/24/2024

Electronic publication
