# Supplementary material for: Combination of locoregional radiotherapy with a TIM-3 aptamer improves survival in diffuse midline glioma models
Source: JCI Insight. 2024 Aug 15;9(18):e175257. doi: 10.1172/jci.insight.175257 (PMC11457855; doi:10.1172/jci.insight.175257)
Supplement: Supplemental data [file jciinsight-9-175257-s270.pdf]

Supplemental data

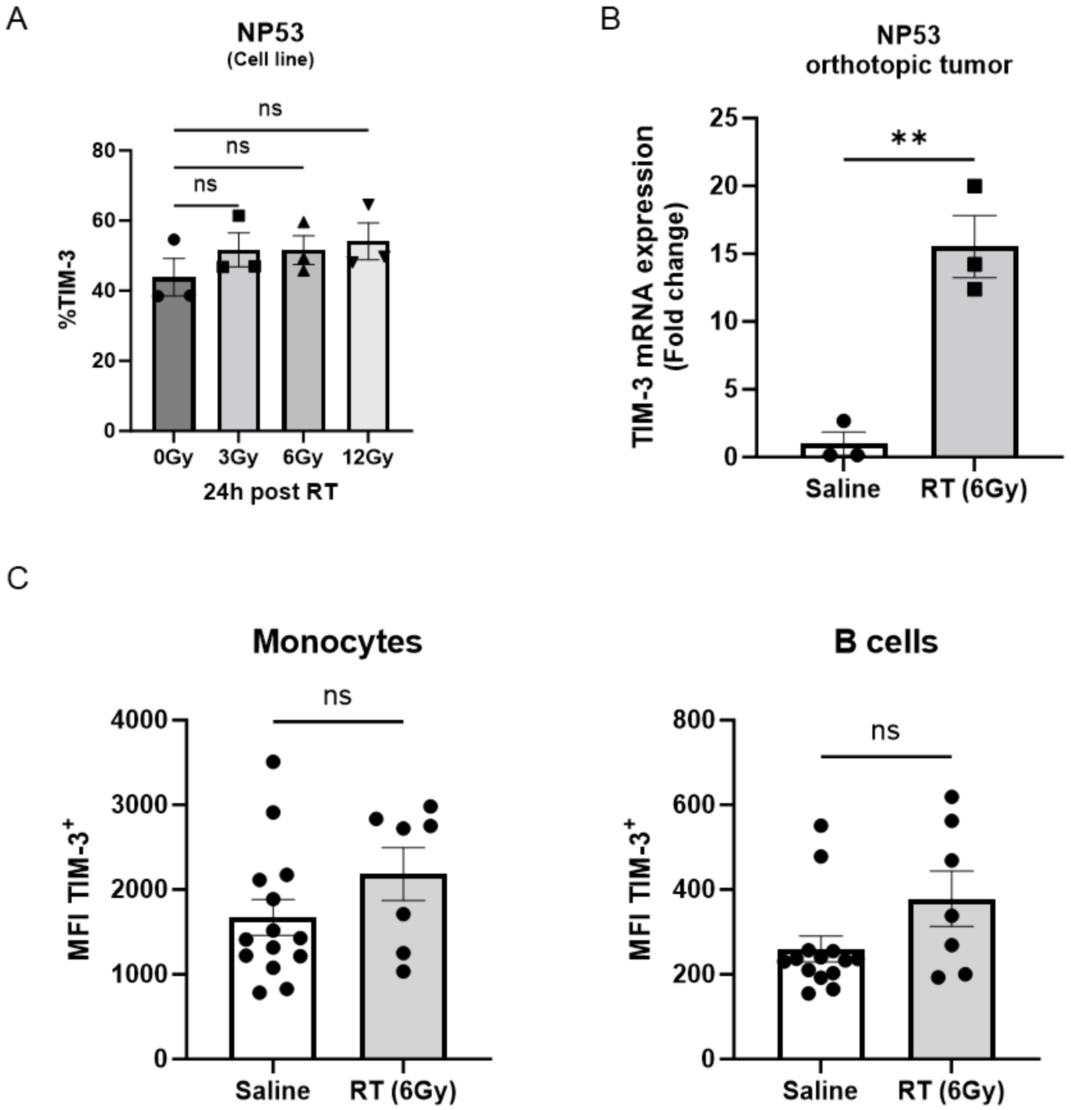

Figure S1: Evaluation of TIM-3 expression after radiotherapy in DMG models. (A) Flow cytometry analysis of TIM-3 expression in NP53 tumor cells 24h post 3, 6, and 12Gy of radiotherapy. **(B)** Real-time-PCR analysis of TIM-3 expression in mice bearing NP53 tumor cells comparing saline and radiotherapy groups. **(C)** Flow cytometry analysis of TIM-3 expression MFI on monocytes and B cells after radiotherapy (6Gy) in a DMG orthotopic model. Data were analyzed with One-way ANOVA (A) and student t-test (B-C) Bar graphs indicate the mean ± SEM (ns, $P>0.05$; *$P<0.05$; **$P<0.01$; ***$P<0.001$; ****$P<0.0001$).

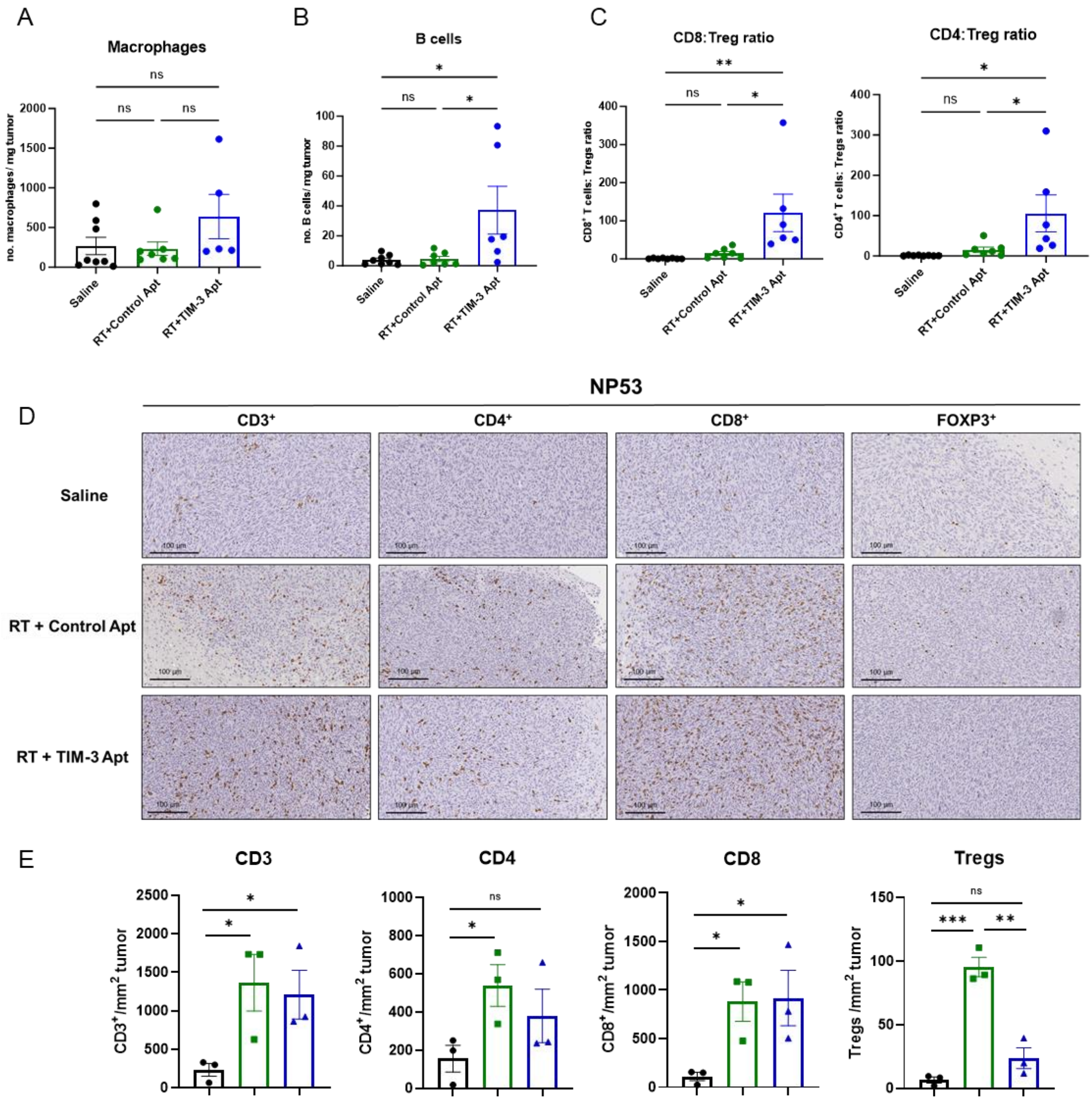

Figure S2: Characterization of inflammatory response produced by radiotherapy and TIM-3 blockade. NP53 cells were engrafted and animals were treated with saline (n=8), radiotherapy (6Gy) control Apt (n=7), or radiotherapy and TIM-3 (n=6). Animals were sacrificed 15 days after the tumor implantation and 8 days after radiotherapy. **(A-B)** Flow cytometry analyses of **(A)** macrophages and **(B)** B cells per mg of tumor on the indicated groups. **(C)** Analyses of the CD8: Treg and CD4: Treg pro-inflammatory ratios in the tumor microenvironment of NP53 mice treated with radiotherapy and TIM-3 Apt. **(D)** CD3, CD4, and CD8 immune-staining representative images from NP53 tumors 15

days after the tumor implantation on indicated treatments. Scale bars: 100µm. **(E)** $CD3^+$, $CD8^+$, $CD4^+$ and Tregs ($FOXP3^+$) cell infiltration per mm^2 of NP53 tumors. Graph showing the quantification of positive cells infiltrating the brain after the indicated treatments per mm^2 (Saline, RT+Control apt, and RT+TIM-3 Apt; n=3-4). Data were analyzed with One-way ANOVA. Bar graphs indicate the mean ± SEM (ns, P>0.05; *P<0.05; **P<0.01; ***P<0.001; ****P<0.0001).

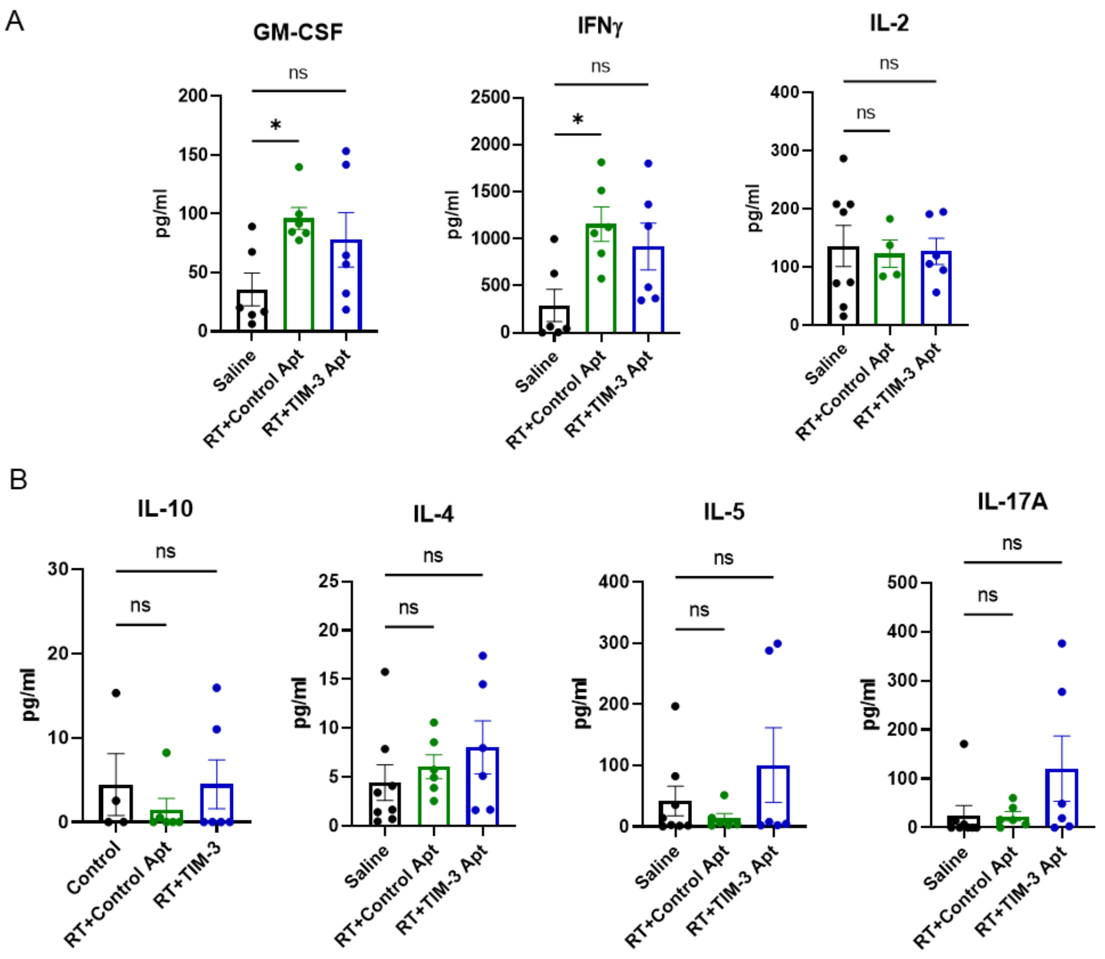

Figure S3: Characterization of inflammatory chemokines and cytokines produced by radiotherapy and TIM-3 blockade. (A) MACSplex analysis in serum of immune innate and Th1 cytokines 15 days after tumor implantation between saline, RT+Control apt, and RT+TIM-3 Apt groups. **(B)** MACSplex analysis in serum of Th2 and Th17 cytokines 15 days after tumor implantation between saline, RT+Control apt, and RT+TIM-3 Apt groups. Data were analyzed with One-way ANOVA. Bar graphs indicate the mean ± SEM (ns, P>0.05; *P<0.05; **P<0.01; ***P<0.001; ****P<0.0001).
